# Intradermal testing increases the accuracy of an immediate-type cefaclor hypersensitivity diagnosis^☆^

**DOI:** 10.1016/j.waojou.2022.100643

**Published:** 2022-04-01

**Authors:** Ji-Hyang Lee, Chan Sun Park, Min Ju Pyo, A. Ryang Lee, Eunyong Shin, Young-Sang Yoo, Woo-Jung Song, Tae-Bum Kim, You-Sook Cho, Hyouk-Soo Kwon

**Affiliations:** a Department of Allergy and Clinical Immunology, Asan Medical Center, University of Ulsan College of Medicine, Seoul, South Korea; b Department of Allergy and Clinical Immunology, Inje University of College of Medicine, Haeundae Paik Hospital, Busan, South Korea

**Keywords:** Beta-lactams, Cephalosporins, Cefaclor, Immediate hypersensitivity, Intradermal skin test, IgE quantification, Drug provocation test, Diagnostic accuracy

## Abstract

**Background:**

Hypersensitivity reactions to cefaclor have increased in accordance with its frequent use. However, only limited data are available on the diagnostic value of skin tests for these conditions, particularly intradermal tests (IDTs).

**Objective:**

To evaluate the clinical usefulness of IDT compared to the ImmunoCAP test in patients with cefaclor-induced immediate-type hypersensitivity.

**Methods:**

We conducted a retrospective chart review from January 2010 to June 2020 of adult subjects from 2 tertiary hospitals in Korea with a history of suspected immediate-type hypersensitivity to cefaclor, and who had undergone ImmunoCAP and IDT.

**Results:**

Overall, 131 subjects diagnosed with cefaclor hypersensitivity were included in the analysis. Fifty-nine patients (59/131, 45.04%) were positive in both IDT and ImmunoCAP. Fifty-four (54/131, 41.22%) and 6 (6/131, 4.58%) subjects showed positive results only with IDT or the ImmunoCAP test, respectively. Twelve subjects (12/131, 9.16%) were negative by both tests but reacted positively in a drug provocation test. The frequency of IDT positivity was similar regardless of the severity of reactions. However, positivity of ImmunoCAP was lower in subjects with mild reactions compared to those with anaphylaxis. Regarding the diagnosis of cefaclor hypersensitivity, the overall sensitivity of IDT and ImmunoCAP was 0.863 and 0.496, respectively while the specificity was 1. The combination of IDT and ImmunoCAP further increased this sensitivity to 0.908.

**Conclusion:**

IDT was more sensitive than ImmunoCAP for the diagnosis of cefaclor allergy, regardless of the severity of the hypersensitivity reaction. Therefore, we recommend a combination of IDT and ImmunoCAP for the diagnosis of cefaclor hypersensitivity.

## Introduction

Allergic reactions to cefaclor, a second-generation cephalosporin antibiotic, are common causes of immunologic drug reactions, and have been recognized since its early clinical use.^1^^,^^2^ Among the hypersensitivity reactions to cefaclor, immediate-type hypersensitivity has been most commonly reported.^3^ Because it is a convenient oral medication, cefaclor is frequently prescribed worldwide and is the second most prescribed systemic antibiotic in Korea.^4^ Accordingly, the number of hypersensitivity reactions to cefaclor has increased over time.^5^

The diagnostic algorithm for beta-lactam hypersensitivity reactions includes a thorough clinical history, skin test, serum drug-specific IgE (sIgE) assay, and drug provocation test (DPT). A cephalosporin skin test, especially an intradermal test (IDT), is considered to have a higher diagnostic value because it has higher sensitivity than a drug-sIgE assay.^6^, ^7^, ^8^ The positive rate of sIgE was relatively high in cases with severe anaphylaxis but not in milder cases.^9^ Therefore, skin testing is still the preferred diagnostic tool for many allergy specialists.

In general, the skin prick test (SPT) is conducted before IDT because it is a simple and safe procedure. However, IDT with a validated nonirritant concentration is the most accurate allergological test for beta-lactams allergy after a DPT. Therefore, when a skin test or *in vitro* test are inconclusive, or when such allergological tests are unavailable, DPTs may be conducted selectively to establish a firm diagnosis.^10^^,^^11^ Despite the high accuracy of a DPT, it can potentially elicit life-threatening reactions and this raises ethical concerns in patients with severe allergic reactions. Therefore, improving the existing allergological tests is essential to reduce the need for potentially risky DPTs. Currently, however, the skin test is standardized mostly for injectable antibiotics because oral antibiotics should undergo complete dissolution and sterilization when used for skin tests.^12^

Among oral cephalosporins, only cefaclor is commercially available for the *in vitro* ImmunoCAP test. However, it is not reliable as a definitive diagnostic tool due to a wide range of sensitivity, which is lower than that of a skin test.^13^, ^14^, ^15^, ^16^ As a consequence, a skin test with cefaclor has frequently been performed by allergy specialists. However, there are only limited data regarding the results of IDT.^7^^,^^12^^,^^16^, ^17^, ^18^, ^19^ Previous larger clinical studies of cefaclor hypersensitivity have mainly utilized SPTs, and the sensitivity of IDT has not been well described, particularly in comparison with ImmunoCAP in subjects with different levels of severity.^13^^,^^20^ We thus aimed to evaluate the clinical value of utilizing both ImmunoCAP and IDT in subjects with cefaclor-induced immediate-type hypersensitivity. We thereby compared the sensitivity of IDT and ImmunoCAP for a diagnosis of cefaclor allergy.

## Methods

### Study subjects

We reviewed subjects (aged 18 years and older) with a history of suspected immediate-type hypersensitivity to cefaclor at 2 tertiary hospitals in Korea from January 2010 through June 2020, who had undergone both ImmunoCAP and IDT. Hypersensitivity to cefaclor was diagnosed when causality assessment was certain or probable according to the World Health Organization (WHO) -Uppsala Monitoring Center criteria and when either cefaclor ImmunoCAP or IDT was positive.^21^ If both allergy tests failed to detect anti-cefaclor IgE despite a highly suggestive clinical history, a DPT was conducted with an incremental dose of cefaclor up to 250 mg without placebo as a comparator. The initial dose was 62.5 mg followed by additional 62.5 mg and 125 mg for most cases with a 30-min interval between doses. Subjects diagnosed with an allergy to other medications taken with cefaclor were excluded from the analyses. A diagnosis of anaphylaxis was based on the diagnostic criteria of the World Allergy Organization (WAO).^22^ The severity of immediate-type hypersensitivity was determined using the classification of Brown et al.^23^ Briefly, a mild reaction indicated only cutaneous/subcutaneous symptoms. A moderate reaction included cardiovascular, respiratory, or gastrointestinal symptoms. Patients presenting with hypoxia, hypotension, or neurologic compromise were defined as having a severe reaction. The moderate to severe systemic reactions are defined by this classification system as anaphylaxis. The study was approved by the Institutional Review Boards of the participating hospitals (IRB No. 2016-0983).

### Detection of total and cefaclor-sIgE in serum

The total IgE and cefaclor-sIgE levels were measured using ImmunoCAP® (Thermo Fisher Scientific Inc., Uppsala, Sweden). A positive cefaclor-sIgE result was defined by a value of 0.35 kU/L or more.

### Intradermal tests

For IDT, cefaclor was prepared as described previously.^18^ Briefly, the powder from a cefaclor capsule was dissolved in 0.9% NaCl solution to a final concentration of 2 mg/mL. IDTs were conducted in 10 healthy subjects to ensure that this concentration did not cause non-specific irritant reactions. All participants showed no erythema or wheals. Based on the nonirritant tests and methods reported in previous studies, a 2 mg/mL concentration was selected as optimal.^16^^,^^18^ This solution was sterilized by filtration through a 0.2 μm syringe filter (Minisarts NML Syringe Filter; Sartorius AG, Goettingen, Germany). The SPT was not routinely conducted ahead of IDT. An intradermal test was performed on the volar forearm with 0.02 mL of the reagent solution. The IDT was considered positive when an increase in the initial wheal diameter of more than 3 mm was accompanied by erythema. Positive controls for IDT were performed with a histamine concentration of 0.1 mg/mL. Normal saline was used as a negative control.

### Statistical analysis

IBM SPSS version 25 for Windows (IBM SPSS Inc., Chicago, IL) was used for statistical analyses. Categorical variables are described as a frequency or proportion, and continuous variables are presented as the mean ± standard deviations. Comparisons between subgroups were conducted using the Student's *t*-test for continuous variables and Pearson's chi-square test or Fisher's exact test for categorical variables. *P*-values less than 0.05 were considered statistically significant.

## Results

### Clinical characteristics of subjects with immediate-type hypersensitivity to cefaclor

**Fig. 1:**
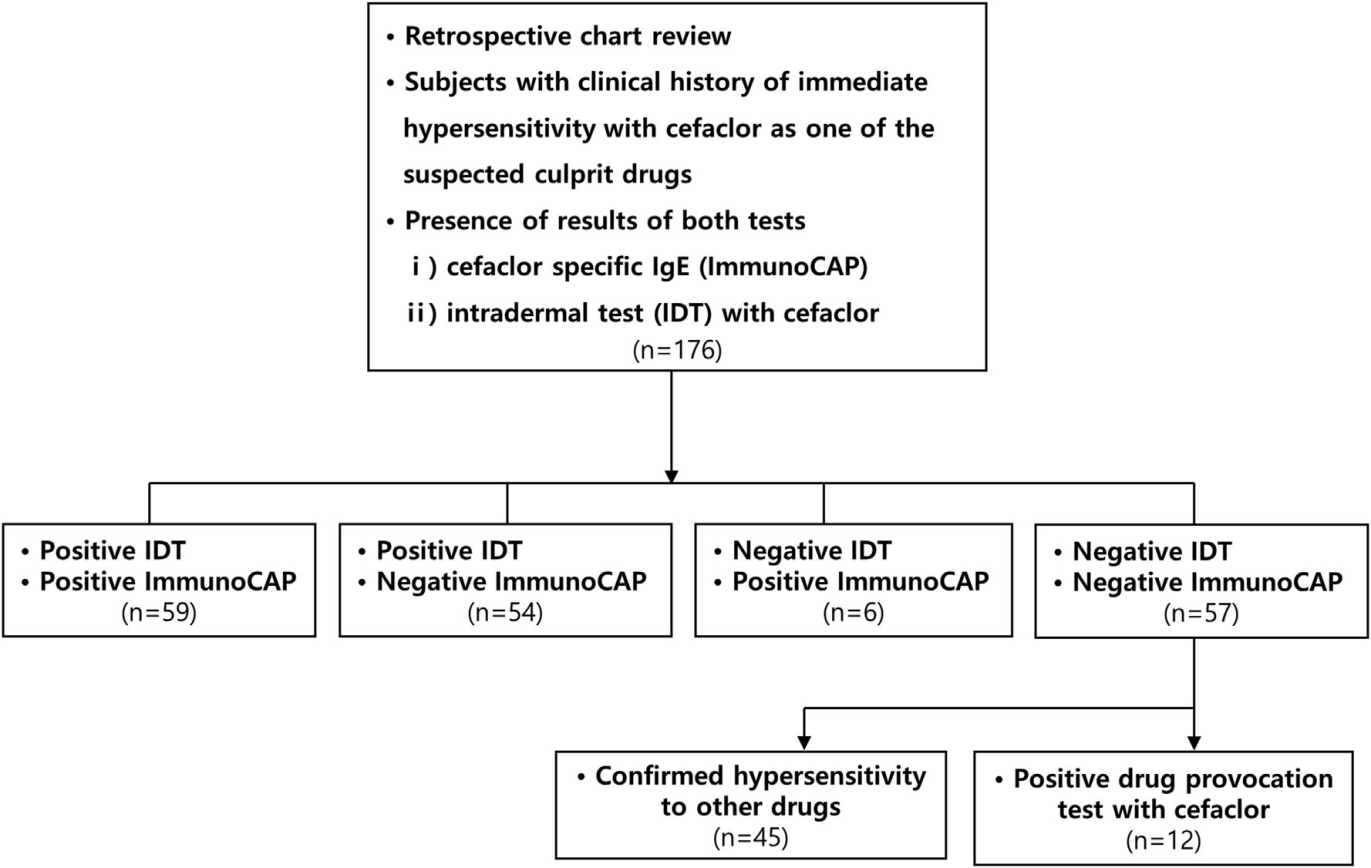
Flow diagram for the inclusion of patients with immediate-type hypersensitivity to cefaclor

The retrospective review identified 176 patients that had undergone both an IDT and cefaclor-sIgE assay because cefaclor was regarded as a candidate causative agent for hypersensitivity reactions. Subjects who showed negative results in both tests were challenged with cefaclor or other suspected drugs to identify the causative agent. Forty-five cases had negative results in both tests but were confirmed to be allergic to other drugs and were therefore excluded from further analysis (Fig. 1). For these patients, the most common causes were non-steroidal anti-inflammatory drugs (NSAIDs) (19/45, 42.22%), followed by histamine H2-antagonists (10/45, 22.22%), and other drugs. The calculated Naranjo adverse drug reaction probability score was also significantly lower in the excluded participants compared to those diagnosed with cefaclor hypersensitivity (6.16 ± 1.06 vs. 8.74 ± 1.17, *P* < 0.001).^24^

Overall, 131 subjects diagnosed with cefaclor allergy were grouped according to the results of the 2 diagnostic tests. Fifty-nine subjects (59/131, 45.04%) were positive in the IDT and ImmunoCAP tests. Fifty-four (54/131, 41.22%) and 6 (6/131, 4.58%) patients had positive results only to IDT or ImmunoCAP, respectively. Twelve cases (12/131, 9.16%) were initially negative to both allergy tests but reacted positively to DPTs conducted later (Table 1). Among the 113 patients with positive IDT, 9 underwent a preceding SPT of which 2 showed a negative result. During IDT, 3 (3/113, 2.65%) patients developed allergic reactions, all of which were managed with medical treatment. Regarding DPT, all the positive cases also improved with either anti-histamine or systemic corticosteroids.

**Table 1:** Clinical data and allergological test results of 131 subjects with an immediate-type hypersensitivity to cefaclor.

Characteristics	IDT+/CAP+	IDT+/CAP–	IDT–/CAP+	IDT–/CAP–	*P* value
	(n = 59)	(n = 54)	(n = 6)	(n = 12)	
Age (y)	46.73 ± 13.05	40.48 ± 14.17	44.83 ± 13.59	38.92 ± 11.35	0.060
Female	35 (59.32)	41 (75.93)	3 (50)	7 (58.33)	0.173
Allergic diseases^a^	23 (38.98)	17 (31.48)	1 (16.67)	2 (16.67)	0.432
Onset time (min)	28.08 ± 43.59	51.09 ± 81.69	23.0 ± 22.80	45.83 ± 36.86	0.260
Interval between event and tests (months)	4.37 ± 9.76	3.33 ± 4.78	2.67 ± 1.86	6.34 ± 7.91	0.622
Severity					
Mild (skin only)	8 (13.56)	19 (35.19)	0	5 (41.67)	0.012
Moderate	31 (52.54)	23 (42.59)	3 (50)	6 (50)	0.765
Severe	20 (33.90)	12 (22.22)	3 (50)	1 (8.33)	0.127
Symptoms					
Cutaneous	58 (98.31)	50 (92.59)	6 (100)	12 (100)	0.449
Respiratory	42 (71.19)	25 (46.30)	6 (100)	7 (58.33)	0.008
Gastrointestinal	22 (37.29)	11 (20.37)	1 (16.67)	3 (25)	0.218
Neurologic	14 (23.73)	3 (5.56)	3 (50)	0	0.002
Cardiovascular	13 (22.03)	8 (14.81)	2 (33.33)	0	0.152
Naranjo scale	8.73 ± 1.19	8.72 ± 1.12	8.83 ± 1.33	8.82 ± 1.40	0.992
Cefaclor-specific IgE (kU/L)	13.01 ± 2.068	0.07 ± 0.10	6.63 ± 7.46	0.02 ± 0.07	<0.001
Total IgE (kU/L)	372.95 ± 423.73	219.15 ± 323.33	255.6 ± 262.82	75.6 ± 57.5	0.109

CAP, ImmunoCAP (cefaclor-sIgE); IDT, intradermal test; IgE, Immunoglobulin E; sIgE, specific IgE.

a Allergic diseases include allergic rhinitis, bronchial asthma, drug allergy, or chronic idiopathic urticaria

The mean ages of these 4 subgroups were similar, ranging from 38.92 to 46.73 years. An overall female predominance was noted but there were no differences in the rate of co-existing allergic diseases such as allergic rhinitis, bronchial asthma, drug allergy, or chronic idiopathic urticaria between the 4 subgroups. The mean onset time of immediate reactions after the intake of cefaclor was less than 60 min, with no significant difference in this duration between the groups. In addition, the time interval between the index event and allergological tests was not significantly different according to the positivity of skin tests or ImmunoCAP.

The severity and manifestation of the allergic reactions were different between patients with different allergy test results (*P* = 0.012). Subjects with a negative IDT and positive ImmunoCAP had more severe reactions and the highest rate of respiratory and neurologic symptoms. By contrast, subjects who had negative results in both tests but had a positive DPT manifested milder reactions with no respiratory or neurologic symptoms. The serum total IgE levels were similar in all groups.

### Results of IDT and ImmunoCAP testing according to the severity of cefaclor hypersensitivity

**Fig. 2:**
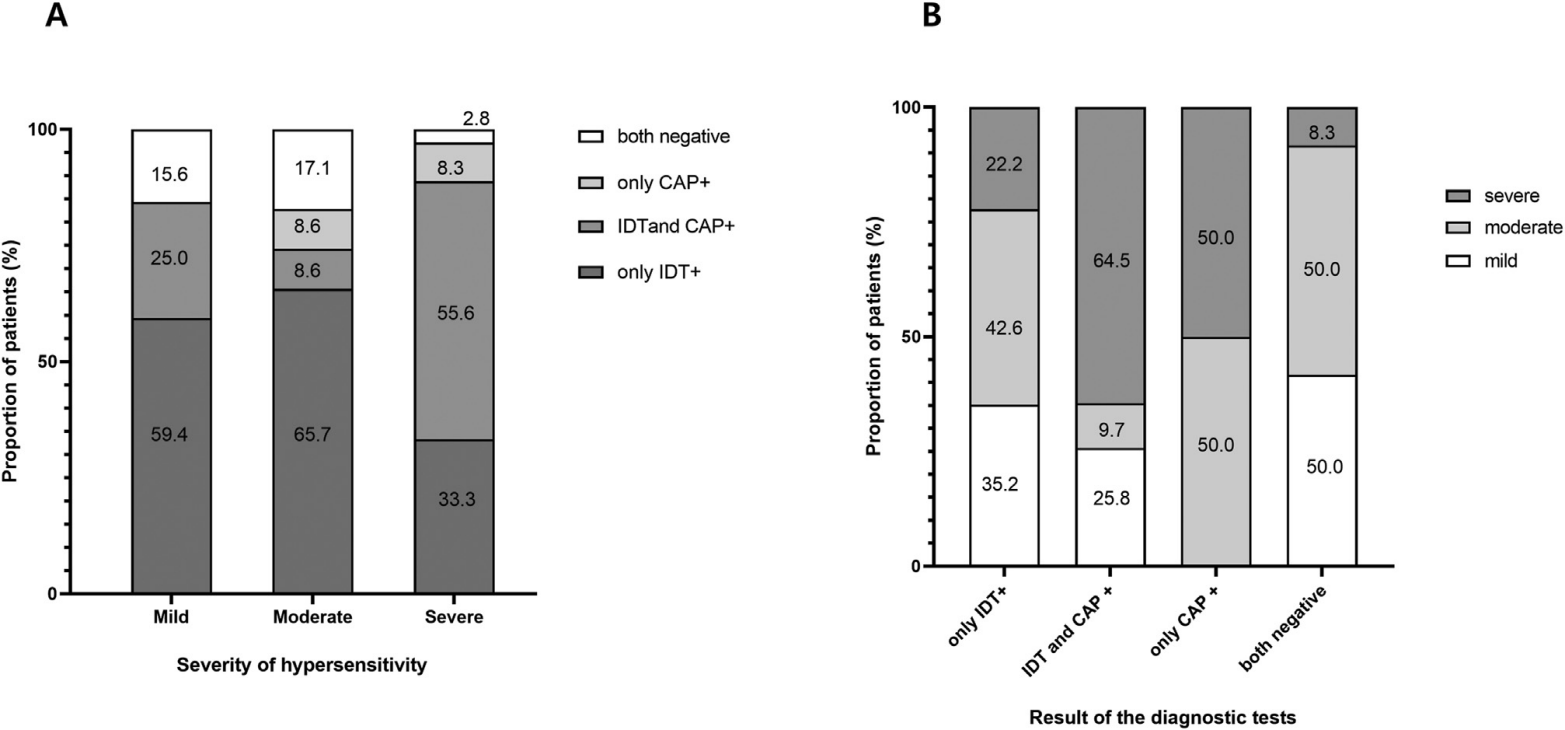
Distribution of patients according to the severity of hypersensitivity (A) and the results of diagnostic tests (B). The numbers inside the boxes are the percentage of patients in each group. CAP, ImmunoCAP (cefaclor-sIgE); IDT, intradermal test

The study subjects were compared according to the severity of their hypersensitivity reactions (Table 2 and Fig. 2). Anaphylaxis was defined as a moderate to severe systemic immediate reaction. The onset time was significantly longer in subjects manifesting mild reactions than in anaphylactic subjects (72.69 ± 102.04 vs. 29.92 ± 39.66, *P* = 0.001). Although the frequency of IDT positivity was similar irrespective of severity, positivity to ImmunoCAP (cut-off value ≥ 0.35 kU/L) was higher in subjects with anaphylaxis compared to those with mild cases (25% vs. 57.58%, *P* = 0.001). Notably however, there were no differences in the levels of sIgE to cefaclor, whereas the total serum IgE level was higher in anaphylactic subjects (149.53 ± 162.45 vs. 325.78 ± 409.58, *P* = 0.032).

**Table 2:** IDT and ImmunoCAP results according to the severity of the immediate-type hypersensitivity to cefaclor.

Characteristics	Mild (skin only) (n = 32)	Anaphylaxis (n = 99)	*P* value
Onset (min)	72.69 ± 102.04	29.92 ± 39.66	0.001
Positive IDT	27 (84.38)	86 (86.87)	0.722
Positive CAP	8 (25)	57 (57.58)	0.001
Cefaclor-sIgE (kU/L)	2.05 ± 6.23	7.53 ± 17.02	0.078
Serum total IgE (kU/L)	149.53 ± 162.45	325.78 ± 409.58	0.032

CAP, ImmunoCAP (cefaclor-sIgE); IDT, intradermal test; IgE, Immunoglobulin E; sIgE, specific IgE

### A higher sensitivity was obtained with a combination of the IDT and ImmunoCAP tests

A recent study reported that an sIgE to cefaclor cut-off value of 0.11 kU/L was optimal for identifying subjects with a cefaclor allergy.^20^ The sensitivity of IDT and ImmunoCAP were analyzed using different sIgE cut-off values. The overall sensitivity of IDT was 0.863 with a specificity of 1. The sensitivity of ImmunoCAP using cut-off values of ≥0.11 and ≥ 0.35 was 0.641 and 0.496, respectively, whereas the specificity of 1 was unchanged. When IDT was utilized in conjunction with ImmunoCAP however (cut-off value ≥ 0.35), the sensitivity increased to 0.908. The combination of these allergy tests using a cut-off value of 0.11 further increased the sensitivity to 0.916 (Table 3).

**Table 3:** Sensitivity of utilizing both IDT and ImmunoCAP with different cut-off values for sIgE to cefaclor in 131 study subjects with cefaclor allergy.

	Sensitivity
IDT only	0.863
CAP only (≥0.11)	0.641
IDT with CAP (≥0.11)	0.916
CAP only (≥0.35)	0.496
IDT with CAP (≥0.35)	0.908

CAP, ImmunoCAP (sIgE to cefaclor); IDT, intradermal test

## Discussion

The present study demonstrated that IDT was more sensitive than the ImmunoCAP test for detecting immediate hypersensitivity to cefaclor. The positivity of IDT was persistently high in both the mild and anaphylactic cases, whereas the positive rate of ImmunoCAP varied significantly according to the severity of the reactions. Therefore, IDT could play an important role in diagnosing cefaclor allergy in cases with a mild reaction, among whom only 25% showed positivity with the ImmunoCAP test. Therefore, utilizing IDT in addition to ImmunoCAP can improve the accuracy of diagnosing a cefaclor allergy, particularly in patients with mild reactions. In addition, because many cases of severe anaphylaxis are negative by ImmunoCAP, we recommend cefaclor-IDT regardless of the severity of the hypersensitivity reactions.

Cefaclor allergy is clinically important as it often presents as anaphylaxis.^14^^,^^15^ Although the precise prevalence is unknown, cefaclor was reported as the most common cause of anaphylaxis in a single referral hospital in Korea.^25^ This underscores the necessity of a precise diagnosis to avoid additional exposure to cefaclor. However, the early confirmation of cefaclor as a causative agent of allergic reactions is challenging because it is often prescribed with NSAIDs and histamine H2-antagonists, which can also induce allergic reactions.^26^^,^^27^ Cefaclor is the only cephalosporin whose sIgE can be measured using a commercial kit, ImmunoCAP. Despite several advantages of this *in vitro* test, the positivity rate of ImmunoCAP in patients with cefaclor was reported to be in the range 25%–89.4%.^13^, ^14^, ^15^, ^16^ Thus, sIgE assays are mainly indicated in addition to a skin test in these patients to reduce the necessity for a DPT, as recently proposed in a European Academy of Allergy and Clinical Immunology position paper.^11^

Among the currently available allergological tests, skin tests are considered to have higher diagnostic value than sIgE assays. Indeed, many prior studies have shown that drug-sIgE assays are less sensitive than skin tests for assessing immediate-type hypersensitivity to beta-lactams.^6^^,^^7^^,^^28^ However, IDTs are generally limited to injectable drugs.^11^ Oral beta-lactams such as amoxicillin and cefaclor may require thorough validation to rule out reactions caused by excipients. Nevertheless, nonspecific irritant reactions or excipient allergies are rare and the clinical use of cefaclor skin tests are well documented.^7^^,^^12^^,^^16^, ^17^, ^18^, ^19^

Novembre and colleagues performed allergological workups in 8 children with a cefaclor allergy using SPTs, IDTs, and ImmunoCAP with a conventional nonirritant concentration of 2 mg/mL. The SPTs yielded a positivity rate of 12.5%, and when performed at a higher concentration of 50 mg/mL, this increased to 75%. When the IDTs were performed at a 2 mg/mL concentration, the final positivity increased to 100%. However, the positivity of ImmunoCAP was only 25% in the same group of children in that study.^16^ Recently, Romano and colleagues also reported a significantly higher positivity with IDT compared to SPT in 19 patients with a cefaclor allergy, along with a low positive rate when using ImmunoCAP.^7^ In line with these previous studies, we also noted a higher sensitivity of a cefaclor IDT compared to ImmunoCAP. Our study included 131 subjects who had ImmunoCAP and IDT results, which is the largest population tested with cefaclor IDT thus far. Moreover, we assumed that positivity rate of skin tests was higher in cefaclor hypersensitivity compared to other injectable beta-lactam antibiotics.^28^^,^^29^

Recently, Nam et al reported that a cut-off level of 0.11 kU/L had greater sensitivity for detecting cefaclor-induced immediate hypersensitivity.^20^ Among the 193 subjects with cefaclor allergy in that study, 9.5%, 60.3%, and 30.2% had mild, moderate, and severe reactions, respectively. When a cut-off of 0.35 kU/L was applied, the sensitivity was 0.709. However, when this cut-off was lowered to 0.11 kU/L, the sensitivity increased to 0.802. In our study, the sensitivity was 0.496 (65/131) when a conventional cut-off of 0.35 kU/L was applied but increased to 0.641 (84/131) using the lower cut-off value of 0.11 kU/L. Nevertheless, the sensitivity of ImmunoCAP with a lower cut-off value was still inferior to that of IDT. The notable discrepancy in the sensitivity calculations between our report and that of Nam et al could be attributable to the higher proportion of subjects with mild reactions in our study population. Our subjects with mild reactions showed a lower rate of ImmunoCAP positivity than those with anaphylaxis, as previously reported.^9^

The epitopes of cephalosporins consist of a chemical structure derived from cephalosporin-protein linkage. The conjugation of cephalosporins to proteins results in cephalosporoyl formation through an amide bond whose unstable structure generates different degradation products that are not readily identifiable, which makes it difficult to fully elucidate the cephalosporin-immunogenicity mechanisms.^30^ This lack of knowledge regarding the epitopes has hindered the further development and improvement of *in vitro* assays. A thorough study of the drug degradation pathways will help identify the intermediate chemical structures of the epitopes and their immunologic recognition is required to eventually improve the sensitivity of drug-sIgE assays.

Alpha-aminocephalosporins, such as cefaclor and cephalexin, have amino groups at their R1 side chain, which are involved in reactions, leading to pyrazinone derivatives. ImmunoCAP utilizes an IgE against an immunoreactive synthetic pyrazinone analogue.^31^ Recently, a new pyrazinone analogue was synthesized and immunologically evaluated, and shown to better mimic the fundamental antigenic determinant than the previously known pyrazinone structure.^19^ In that same study, the sera from 8 cefaclor allergy subjects with a positive skin test were used to compare the immunological recognition of the epitopes. Sera from 5 of 8 subjects showed meaningful recognition of the novel pyrazinone analogue, whereas only 1 serum sample recognized the previously mentioned structure. Interestingly, the sera from 2 of these subjects reacted positively to an SPT but did not recognize either of the pyrazinone analogues. Such data indicate there are diverse immunoreactive cefaclor epitopes that are not yet known to be detected by an sIgE and which are only identifiable by a skin test or DPT.

We speculate that there may also be heterogeneity in the avidity of sIgE against immunoreactive epitopes. The mean onset time was 29.9 min in the anaphylaxis group, the members of which showed an ImmunoCAP positivity of 57.6%. However, the mean onset time of mild reactions was 72.7 min, markedly longer than that for the usual immediate-type reactions, and the ImmunoCAP test was positive in only 25% of these cases. Therefore, some immunoreactive products other than pyrazinone structures may elicit IgE reactions with very low avidity. When challenged systemically, some epitopes may require a longer time to be degraded and accumulated at sufficient levels within tissues to elicit clinical symptoms. In contrast, IDT introduces a high concentration of the causative drug to a limited tissue area where degraded epitopes quickly reach the threshold to evoke wheal and flare reactions within 20 min. In any case, an IDT may have a higher sensitivity by reacting to diverse immunoreactive cefaclor epitopes whereas ImmunoCAP recognizes only a specific pyrazinone structure.^19^^,^^32^

Our study had several limitations. First, retrospective inclusion of only subjects who received both an IDT and ImmunoCAP test may have resulted in selection bias. The clinicians treating these cases could have prescribed both allergological tests because of a high suspicion of cefaclor hypersensitivity and requirement for a thorough work-up. A second limitation is that cefaclor is a noninjectable preparation containing possible non-dissolved particulates, which might act as irritants. To minimize irritation from particulates or microorganisms in the capsule powder, we filtered these solutions through a 0.2-μm syringe. Third, the ingredients of the cefaclor capsule include excipients that could potentially act as allergens or activate mast cells. However, we and others have conducted IDTs with 2 mg/mL concentrations in healthy subjects and observed no irritant reactions.^16^^,^^18^ Moreover, the cefaclor preparation ingredients include hydroxypropyl cellulose (low-substituted), dimethicone, pregelatinized starch, sodium starch glycolate, and magnesium stearate, which rarely cause allergies.^33^ Last, a diagnosis of cefaclor allergy was not confirmed by DPT in all cases, which needs to be considered when interpreting the diagnostic value of IDT and ImmunoCAP. Since this was a retrospective study reflecting real-world practice, physicians did not proceed with an additional DPT when cefaclor allergy was strongly suspected by history, and either a skin test or ImmunoCAP supported this suspicion. This approach was consistent with real-world clinical practices by allergy specialists in other countries.^34^ In our data, more than half of patients (70/131, 53.44%) experienced multiple hypersensitivity reactions only with cefaclor. In addition, simultaneous conducted allergological tests (including DPT) with other medications taken together with cefaclor reported negative results. This was reflected as a high Naranjo score (8.74 ± 1.17) indicating definite probability.

Notwithstanding the aforementioned limitations, we have provided robust evidence that IDT is more sensitive than ImmunoCAP for detecting immediate hypersensitivity to cefaclor. Unlike the ImmunoCAP test, in which positivity is largely dependent on the severity of the hypersensitivity reaction, the positive rate with IDT was high (approximately 85%) irrespective of severity. For the management of drug-induced hypersensitivity, the identification of the causative agent is an essential task for allergists. Considering the increasing use of oral beta-lactams and expected higher burden of hypersensitivity reactions, a more accurate and standardized diagnostic protocol is required. In this regard, we recommend conducting IDT with ImmunoCAP for the diagnosis of cefaclor hypersensitivity.

## Abbreviations

sIgE, specific IgE; DPT, drug provocation test; IDT, intradermal test; SPT, skin prick test; NSAIDs, non-steroidal anti-inflammatory drugs.

## Funding

None.

## Availability of data and materials

The datasets analyzed during the current study are available from the corresponding author on reasonable request.

## Authors’ contributions

Study design: HSK and YSC, data collection: CSP, ES, YSY, WJS, TBK, HSK, YSC, data analysis: JHL, MJP, ARL, writing the manuscript: JHL and CSP, reviewing the manuscript: WJS, TBK, HSK, YSC, final approval of the manuscript: all authors.

## Ethics approval

The present study was reviewed and approved by the Institutional Review Board of Asan Medical Center (IRB No. 2016-0983).

## Authors’ consent for publication

All authors have agreed with the publication of this work in World Allergy Organization Journal.

## Submission declaration

This manuscript has not been published or presented elsewhere in part or in entirety and is not under consideration by another journal.

## Declaration of competing interest

None to declare.

## References

[1] Lovell S.J., Reid W.D. (1982). Serum sickness with cefaclor. Can Med Assoc J.

[2] Murray D.L., Singer D.A., Singer A.B., Veldman J.P. (1980). Cefaclor–a cluster of adverse reactions. N Engl J Med.

[3] Hama R., Mori K. (1988). High incidence of anaphylactic reactions to cefaclor. Lancet.

[4] Sohn H.S., Oh O.H., Kwon J.W., Lee Y.S. (2013). Higher systemic antibiotic consumption in a population of South Korea (2008–2009). Int J Clin Pharmacol Therapeut.

[5] Atanaskovic-Markovic M., Velickovic T.C., Gavrovic-Jankulovic M., Vuckovic O., Nestorovic B. (2005). Immediate allergic reactions to cephalosporins and penicillins and their cross-reactivity in children. Pediatr Allergy Immunol.

[6] Mayorga C., Celik G., Rouzaire P. (2016). In vitro tests for drug hypersensitivity reactions: an ENDA/EAACI drug allergy interest group position paper. Allergy.

[7] Romano A., Valluzzi R.L., Caruso C., Zaffiro A., Quaratino D., Gaeta F. (2021). Evaluating immediate reactions to cephalosporins: time is of the essence. J Allergy Clin Immunol Pract.

[8] Mirakian R., Leech S.C., Krishna M.T. (2015). Management of allergy to penicillins and other beta-lactams. Clin Exp Allergy.

[9] Fontaine C., Mayorga C., Bousquet P.J. (2007). Relevance of the determination of serum-specific IgE antibodies in the diagnosis of immediate beta-lactam allergy. Allergy.

[10] Macy E., Romano A., Khan D. (2017). Practical management of antibiotic hypersensitivity in 2017. J Allergy Clin Immunol Pract.

[11] Romano A., Atanaskovic-Markovic M., Barbaud A. (2020). Towards a more precise diagnosis of hypersensitivity to beta-lactams – an EAACI position paper. Allergy.

[12] Brockow K., Garvey L.H., Aberer W. (2013). Skin test concentrations for systemically administered drugs – an ENDA/EAACI drug allergy interest group position paper. Allergy.

[13] Rhyou H.I., Doo G.E., Yoon J. (2021). Clinical characteristics and risk factors for cefaclor-induced immediate hypersensitivity: a retrospective observation at two university hospitals in Korea. Allergy Asthma Clin Immunol.

[14] Nam Young-Hee, Kim Jeong Eun, Hwang Eui-Kyung (2011). Clinical and immunologic evaluations of immediate hypersensitivity to cefaclor. Korean J Asthma Allergy Clin Immunol.

[15] Yoo H.S., Kim S.H., Kwon H.S. (2014). Immunologic evaluation of immediate hypersensitivity to cefaclor. Yonsei Med J.

[16] Novembre E., Mori F., Pucci N., Bernardini R., Romano A. (2009). Cefaclor anaphylaxis in children. Allergy.

[17] Romano A., Gaeta F., Valluzzi R.L., Alonzi C., Viola M., Bousquet P.J. (2008). Diagnosing hypersensitivity reactions to cephalosporins in children. Pediatrics.

[18] Romano A., Gueant-Rodriguez R.M., Viola M. (2005). Diagnosing immediate reactions to cephalosporins. Clin Exp Allergy.

[19] Martin-Serrano A., Mayorga C., Barrionuevo E. (2020). Design of an antigenic determinant of cefaclor: chemical structure-IgE recognition relationship. J Allergy Clin Immunol.

[20] Nam Y.H., Lee S.H., Rhyou H.I. (2018). Proper cut-off levels of serum specific IgE to cefaclor for patients with cefaclor allergy. Yonsei Med J.

[21] Edwards I.R., Aronson J.K. (2000). Adverse drug reactions: definitions, diagnosis, and management. Lancet.

[22] Cardona V., Ansotegui I.J., Ebisawa M. (2020). World allergy organization anaphylaxis guidance 2020. World Allergy Organ J.

[23] Brown S.G. (2004). Clinical features and severity grading of anaphylaxis. J Allergy Clin Immunol.

[24] Naranjo C.A., Busto U., Sellers E.M. (1981). A method for estimating the probability of adverse drug reactions. Clin Pharmacol Ther.

[25] Seo Yuri, Han Yeseul, Kim Soo Hyun (2017). Clinical features of serious adverse drug reactions in a tertiary care hospital in Korea. Korean J Med.

[26] Park K.H., Pai J., Song D.G. (2016). Ranitidine-induced anaphylaxis: clinical features, cross-reactivity, and skin testing. Clin Exp Allergy.

[27] Kowalski M.L., Makowska J.S., Blanca M. (2011). Hypersensitivity to nonsteroidal anti-inflammatory drugs (NSAIDs) – classification, diagnosis and management: review of the EAACI/ENDA(#) and GA2LEN/HANNA. Allergy.

[28] Sousa-Pinto B., Tarrio I., Blumenthal K.G. (2021). Accuracy of penicillin allergy diagnostic tests: a systematic review and meta-analysis. J Allergy Clin Immunol.

[29] Joerg L., Hasler S., Gschwend A. (2021). 75% negative skin test results in patients with suspected hypersensitivity to beta-lactam antibiotics: influencing factors and interpretation of test results. World Allergy Organ J.

[30] Perez-Inestrosa E., Suau R., Montanez M.I. (2005). Cephalosporin chemical reactivity and its immunological implications. Curr Opin Allergy Clin Immunol.

[31] Venemalm L. (2001). Pyrazinone conjugates as potential cephalosporin allergens. Bioorg Med Chem Lett.

[32] Kim S.H., Choi J.H., Park H.S. (2005). Heterogeneity of the IgE response to allergenic determinants of cefaclor in serum samples from patients with cefaclor-induced anaphylaxis. Ann Allergy Asthma Immunol.

[33] Reker D., Blum S.M., Steiger C. (2019). "Inactive" ingredients in oral medications. Sci Transl Med.

[34] Torres M.J., Celik G.E., Whitaker P. (2019). A EAACI drug allergy interest group survey on how European allergy specialists deal with beta-lactam allergy. Allergy.

